# German Hypothermia Registry Improves Clinician Confidence and Harmonises Decentralised Neonatal Hypoxic‐Ischaemic Encephalopathy Care in Germany

**DOI:** 10.1111/apa.70579

**Published:** 2026-05-08

**Authors:** Till Dresbach, Khaled Abdelrahman, Sebiha Demir, Anne Groteklaes, Alexandra Boeckenhoff, Andreas Mueller, Hemmen Sabir, the German Hypothermia Registry, Milian Brasche, Nicolas Allgaier, Daniel Klotz, Norbert Teig, Katja Schneider, Hemmen Sabir, Sandra Holz, Birte Tröger, Thorsten Körner, Uwe Höpner, Marie Schmenk, Michael Hofmann, Mario Rüdiger, Julia Osterholt, Martin Berghäuser, Christopher Willerscheidt, Farina Morgenroth, Hans Jörg Bittrich, Steven Hébert, Arber Arapi, Anja Stein, Veit Lange, Gunnar Rau, Nikolas Hillenbrandt, Matthias Reger, Susanne Schade, Jana Dieks, Heimke von Osten, Marita Horstkemper, Martin Blohm, Mario Lange, Bettina Bohnhorst, Simon Kranig, Maurice Petrasch, Nasenien Nourkami‐Tutdibi, Christiane Pinkwart, Thomas Kriebel, Julia Jerabek, Nora Bonney, Angela Kribs, Clemens Andrée, Sophia Stadermann, Kirsten Glaser, Peter Jahn, Katrin Braun, Anna Eisenhardt, Alba Perez‐Ortiz, Nadine Mand, Timea Takacs, Anja Jäger, Oliver Schroers, Esther Schouten, Claudia Nussbaum, Kai Böckenholt, Rüdiger Wentzell, Guido Engelmann, Katherine Louise Kastor, Michael Schroth, Matthias Engler, Friederike Eichhorn, Moritz Rohrbach, Fabian Bärtling, Frank Dohle, Matthias Keller, Marion Weber, Verena Lehnerer, Anja Mayer, Marie‐Claire Detemple, Valentin Oellers, Markus Pingel, Friederike Eilbracht, Jutta Adler, Elena Eberle, Karoline Goj, Virginia Toth, Andrea Czoske, Cornelia Wiechers, Christiane Mayer, Anja Schumann, Patrick Salaschek, Janaina Rauch, Kai Hensel, Pia Paul

**Affiliations:** ^1^ Department of Neonatology and Paediatric Intensive Care Medicine University Hospital Bonn Bonn Germany

**Keywords:** amplitude‐integrated electroencephalography, German hypothermia registry, hypoxic‐ischaemic encephalopathy, neurological assessment, web‐based survey

## Abstract

**Aim:**

Decentralised implementation of therapeutic hypothermia (TH) for neonatal hypoxic‐ischaemic encephalopathy (HIE) in Germany historically caused heterogeneous clinical practices. The aim of this study was to assess the impact of the German Hypothermia Registry on clinical standardisation and confidence in neonatal HIE care since its initiation in 2022.

**Methods:**

We conducted a nationwide, two‐phase, web‐based survey in 2022 (*n* = 66) and 2025 (*n* = 222). A longitudinal subgroup analysis included physicians participating in both surveys (*n* = 30). Outcomes included guideline adherence, subjective diagnostic and therapeutic confidence, and perceived needs for further standardisation.

**Results:**

From 2022 to 2025, clinician confidence improved across most domains. Complete confidence in diagnosing HIE increased from 42.4% to 80.0% in the longitudinal subgroup, while confidence in performing TH rose from 53.0% to 86.7%. Clarity regarding inclusion criteria reached 100% among longitudinal participants. In contrast, complete confidence in neurological assessment (36.7%) and amplitude‐integrated electroencephalography (aEEG) interpretation (60.0%) remained comparatively lower in 2025.

**Conclusion:**

The German Hypothermia Registry markedly improved standardisation and clinician confidence in neonatal HIE care. However, neurological assessment and aEEG interpretation remain key areas requiring targeted educational support.

## Summary

- As decentralised care for neonatal hypoxic‐ischaemic encephalopathy in Germany caused inconsistent practices, the German Hypothermia Registry was launched to establish a unified national approach.
- Between 2022 and 2025, continuous Registry participation led to substantial improvement in protocol adherence and strengthened clinicians' confidence in the diagnosis and performance of therapeutic hypothermia.
- Despite therapeutic advances, clinical neurological assessment and interpretation of amplitude‐integrated electroencephalography remain a challenge, highlighting the need for targeted educational efforts.

**Abbreviations**
aEEG: amplitude‐integrated electroencephalography
HIE: hypoxic–ischaemic encephalopathy
NICU: neonatal intensive care unit
TH: therapeutic hypothermia

## 1 Introduction

Rare diseases pose a particular challenge to healthcare systems, as individual centres often lack standardised routines due to the low number of cases. This has subsequently led to heterogeneous and inconsistent care [1]. Hypoxic‐ischaemic encephalopathy (HIE) is a prime example of how neonatal and long‐term neurodevelopmental outcomes are strictly dependent on rapid and standardised intervention [2]. Despite medical and technological advances in high‐income countries, HIE still remains a critical condition, affecting approximately 1–3 per 1000 live births each year [3, 4, 5]. While therapeutic hypothermia (TH) is the established standard treatment of moderate to severe HIE, its implementation in Germany has been notably decentralised [6, 7, 8]. In contrast to many neighbouring European countries, TH is rarely centralised at regional centres in Germany. Instead, the German Federal Joint Committee allows almost all higher level neonatal intensive care units (NICUs) with appropriate technology to not only initiate but also conduct this therapy [9, 10, 11].

Before a national Registry for HIE and TH was established, initial assessments revealed a significant lack of consensus on key management aspects. In Germany, Level 1 perinatal hospitals represent the highest category of national neonatal care and structurally correspond to international/AAP Level III and IV units. A landmark 2019 survey of physicians at 107 of these hospitals highlighted wide variations in clinical practice. The survey noted that 70.0% of participating centres treated fewer than five infants with TH per year, which resulted in limited experience at individual hospitals. Furthermore, discrepancies were found in almost every aspect of care. Regarding supportive treatment strategies, only 30.0% of units primarily ventilated infants during cooling. Enteral feeding practices also varied widely and ranged from full oral feeding to trophic feeding. Finally, the timing of diagnostic neuroimaging ranged from the first days of life to several months post‐discharge [12].

To bridge this gap and establish a unified national approach, the German Hypothermia Registry was launched at the end of 2022. Initially involving 10 maximum‐care NICUs/AAP Level III and IV units, the Registry aimed to actively shape clinical practice through harmonisation and education. Over the past 3 years, it has evolved into a nationwide professional network with its efforts extending far beyond data collection. The German Hypothermia Registry facilitates regular clinical teachings, interdisciplinary expert exchange, and standard operating procedures adapted from international guidelines [10].

By 2025, the impact of these efforts has become evident. The Registry expanded to include 80 NICUs, predominantly German Level 1 perinatal hospitals, across the country. A 2025 study of the primary raw data submitted to the Registry, involving 262 infants, systematically identified the leading admission criteria for TH in Germany. It highlighted severe metabolic acidosis and abnormal amplitude‐integrated electroencephalography (aEEG) patterns as the most decisive factors for treatment initiation. In addition, the study validated several clinical assessment tools through Registry data and found the Sarnat score to be the strongest predictor of abnormal aEEG background activity [9].

Despite these advancements and the central conclusion of the 2025 study that early diagnosis is critical, the success of standardised protocols ultimately depends on clinician confidence and adherence to these protocols. This study aims to quantify the impact of the Registry's activities on clinical care before and 3 years after the implementation of nationwide harmonisation and teaching efforts. Using a dual‐survey approach conducted in 2022 and 2025, we compared subjective confidence, diagnostic skills and perceived need for further standardisation. The study further seeks to identify remaining challenges by pinpointing remaining gaps in knowledge, barriers in interpretation of diagnostic tools or persistent uncertainties in communication that may require further targeted interventions.

## 2 Material & Methods

### 2.1 Study Design and Setting

This was a nationwide, longitudinal, web‐based survey that used the framework of the German Hypothermia Registry. A two‐phase survey approach was used to assess the impact of the Registry's educational initiatives, standardised protocols, and professional exchange formats. The first survey was administered at the initiation of the Registry in 2022, and the second survey was conducted in 2025 to evaluate its impact after 3 years of nationwide implementation. During this period, the Registry expanded from 10 initial NICUs to 80 participating NICUs across Germany.

### 2.2 Participants and Data Collection

The study population included physicians of all professional levels, ranging from paediatric residents to department chiefs of neonatology and intensive care. The survey was conducted online using the commercial platform SurveyMonkey. The survey link was emailed to the respective contact persons of all participating NICUs of the German Hypothermia Registry, who were instructed to forward the link to their respective physician staff. While participation was voluntary and anonymous, up to three email reminders were sent to the entire mailing list of contact persons for broader participation. The design of the study allowed for both a broad cross‐sectional comparison between survey years and a focused longitudinal subgroup analysis of physicians with sustained Registry involvement over the past 3 years.

### 2.3 Survey Instrument

The survey consisted of 22 items covering three main domains: adherence to clinical guidelines, subjective confidence in diagnostic and therapeutic procedures, and communication and perceived needs for further standardisation and support. Diagnostic confidence was assessed with regard to the criteria for perinatal asphyxia and HIE, as well as aEEG interpretation. Therapeutic confidence included the initiation and performance of TH, seizure management, and short‐term and long‐term post‐discharge follow‐up care. Communication confidence focused on the ability of physicians to provide clear and consistent information to parents regarding treatment, risks, and prognosis. Most items were rated on a four‐point Likert scale ranging from 1 for completely disagree to 4 for completely agree. In addition, demographic data such as professional role, years of experience in neonatology, and annual case volumes were collected. The full survey is presented in Appendix S1.

### 2.4 Cohort Framework and Analysis

To evaluate the overall impact of the Registry, a cross‐sectional comparison was first performed between the complete 2022 cohort of 66 respondents and the complete 2025 cohort of 222 respondents. This analysis aimed to capture changes in clinical confidence and practice patterns following the nationwide expansion of the Registry.

In a second step, a longitudinal subgroup analysis was conducted focusing on the 30 physicians who self‐reported participation (item 22 in Appendix S2) in both survey cycles. By isolating this subgroup, the study aimed to identify effects specifically associated with sustained exposure to the Registry's educational activities and standardised diagnostic and therapeutic tools.

Combining cross‐sectional and longitudinal perspectives allowed us to differentiate between general trends in professional development and a more specific Registry impact. This ultimately provided a comprehensive assessment of the Registry's influence on neonatal HIE care in Germany.

### 2.5 Statistical Analysis

The statistical analysis was descriptive in nature. Survey results are presented as absolute numbers and percentages. No formal statistical testing was performed due to differences in cohort composition between the two survey time points and the ordinal structure of Likert‐scale responses.

### 2.6 Ethics

The study was approved by the local ethics committee of the University of Bonn (nr. 472/22‐E) and was conducted in accordance with its regulations and guidelines.

## 3 Results

### 3.1 Participant Demographics and Professional Experience

We analysed 288 survey responses: 66 in 2022 and 222 in 2025. These responses were provided by 258 unique participants, as 30 physicians participated in both surveys and formed our longitudinal subgroup. The professional composition of the 2022 cohort included 24 paediatric residents (36.4%), 21 fellows in neonatology (31.8%) and 21 consultants in neonatology (31.8%). The 2025 cohort comprised 58 paediatric residents (26.1%), 34 fellows in neonatology (15.3%), 107 consultants in neonatology (48.2%) and 16 department chiefs (7.2%). Within the 2025 cohort, a longitudinal subgroup of participants was identified, of which 17 (56.7%) were consultants and 8 (26.7%) were department chiefs. Regarding work experience in neonatology, 43.9% of the 2022 cohort reported five or more years of experience, whereas this proportion increased to 61.7% in the 2025 cohort and to 93.3% in the longitudinal subgroup. Personal exposure to TH remained regular. In 2022, 71.2% of participants reported managing more than five cases in the preceding 12 months. In 2025, 70.3% of respondents reported managing at least three cases during the same period (Table 1).

**TABLE 1:** Participant characteristics and demographic data.

Characteristic	Cohort 2022 (*n* = 66)	Cohort 2025 (*n* = 222)	Subgroup 2025 (*n* = 30)
**Professional position**			
Paediatric resident	24 (36.4%)	58 (26.1%)	1 (3.3%)
Fellow in neonatology	21 (31.8%)	34 (15.3%)	4 (13.3%)
Consultant in neonatology	21 (31.8%)	107 (48.2%)	17 (56.7%)
Department chief	—	16 (7.2%)	8 (26.7%)
Other	—	5 (2.3%)	0 (0.0%)
**Work experience in neonatology**			
0–1 year	16 (24.2%)	28 (12.6%)	0 (0.0%)
2–4 years	21 (31.8%)	57 (25.7%)	2 (6.7%)
≥ 5 years	29 (43.9%)	137 (61.7%)	28 (93.3%)
**Clinical exposure to TH (last 12 months)**			
0 cases	3 (4.5%)	—	—
1–5 cases	16 (24.2%)	—	—
> 5 cases	47 (71.2%)	—	—
1 case	—	27 (12.2%)	2 (6.7%)
2 cases	—	38 (17.1%)	8 (26.7%)
3 cases	—	43 (19.4%)	6 (20.0%)
4 cases	—	42 (18.9%)	2 (6.7%)
5 cases	—	34 (15.3%)	4 (13.3%)
6 cases	—	13 (5.9%)	3 (10.0%)
≥ 7 cases	—	22 (9.9%)	5 (16.7%)
**Employment status**			
Full‐time	52 (78.8%)	159 (71.6%)	20 (66.7%)
Part‐time	14 (21.2%)	63 (28.4%)	10 (33.3%)

*Note:* The longitudinal subgroup (*n* = 30) consists of physicians who participated in both the 2022 baseline survey and the 2025 follow‐up survey.

### 3.2 Institutional Protocols and Standardised Treatment Recommendations

In 2022, 41 physicians (62.1%) reported that TH was performed according to the national guidelines of the German association of scientific medical societies, while 20 physicians (30.3%) reported the use of clinic‐specific standard operating procedures. By 2025, the general cohort showed 98 physicians (44.1%) following national guidelines and 119 physicians (53.6%) using internal standard operating procedures. In the longitudinal subgroup, 9 physicians (30.0%) followed national guidelines and 21 physicians (70.0%) utilised internal standard operating procedures in 2025. The desire for a national uniform protocol with standardised treatment recommendations was supported by 83.3% of physicians in 2022 and 88.3% in 2025, who indicated they completely agree with this need. Furthermore, 75.8% of the 2022 cohort and 77.9% of the 2025 cohort completely agreed that such standardised recommendations would facilitate their daily work and increase their confidence (Table 2).

**TABLE 2:** Institutional protocols and perceived need for standardisation.

Item	Cohort 2022 (*n* = 66)	Cohort 2025 (*n* = 222)	Subgroup 2025 (*n* = 30)
**Protocol used for TH**			
National guidelines of the German association of scientific medical societies	41 (62.1%)	98 (44.1%)	9 (30.0%)
Clinic‐specific standard operating procedures	20 (30.3%)	119 (53.6%)	21 (70.0%)
Individual treatment instruction by senior colleague	4 (6.1%)	3 (1.4%)	0 (0.0%)
Do not know	1 (1.5%)	1 (0.5%)	0 (0.0%)
**Desire for national uniform protocol**			
Completely agree	55 (83.3%)	196 (88.3%)	26 (86.7%)
Partially agree	10 (15.2%)	20 (9.0%)	4 (13.3%)
Rather/completely disagree	0 (0.0%)	5 (2.3%)	0 (0.0%)
**Standardised recommendations facilitate daily work**			
Completely agree	50 (75.8%)	173 (77.9%)	21 (70.0%)
Partially agree	12 (18.2%)	37 (16.7%)	7 (23.3%)
Rather/completely disagree	4 (6.0%)	9 (4.1%)	2 (6.7%)
**Option for online specialist consultation**			
Completely agree	27 (40.9%)	105 (47.3%)	15 (50.0%)
Partially agree	21 (31.8%)	69 (31.1%)	8 (26.7%)
Rather/completely disagree	18 (27.3%)	47 (21.2%)	7 (23.3%)

*Note:* The longitudinal subgroup (*n* = 30) consists of physicians who participated in both the 2022 baseline survey and the 2025 follow‐up survey.

### 3.3 Diagnostic and Therapeutic Confidence

Regarding the criteria for diagnosing perinatal asphyxia, 66.7% of the 2022 cohort, 75.2% of the 2025 general cohort and 90.0% of the longitudinal subgroup reported feeling completely confident. For the diagnosis of HIE, 42.4% of the 2022 cohort, 45.9% of the 2025 general cohort and 80.0% of the longitudinal subgroup felt completely confident. Clarity regarding inclusion criteria for TH was reported at a high subjective confidence level by 77.3% in 2022 and rose to 86.5% in 2025, whilst reaching 100% within the longitudinal subgroup. Furthermore, complete subjective confidence in the actual performance of TH was stated by 53.0% of the 2022 participants, 71.2% of the 2025 general cohort and 86.7% of the longitudinal subgroup (Table 3).

**TABLE 3:** Diagnostic, therapeutic, and communication confidence.

Confidence domain (completely agree)	Cohort 2022 (*n* = 66)	Cohort 2025 (*n* = 222)	Subgroup 2025 (*n* = 30)
Diagnosis of asphyxia	44 (66.7%)	167 (75.2%)	27 (90.0%)
Diagnosis of HIE	28 (42.4%)	102 (45.9%)	24 (80.0%)
aEEG interpretation	21 (31.8%)	85 (38.3%)	18 (60.0%)
Clarity of TH inclusion criteria	51 (77.3%)	192 (86.5%)	30 (100%)
Indications for TH	37 (56.1%)	139 (62.6%)	23 (76.7%)
Performance of TH	35 (53.0%)	158 (71.2%)	26 (86.7%)
Neurological assessment	14 (21.2%)	65 (29.3%)	11 (36.7%)
Seizure management	37 (56.1%)	148 (66.7%)	24 (80.0%)
Initiation of follow‐up care	24 (36.4%)	107 (48.2%)	20 (66.7%)
Communication with parents^a^	—	133 (59.9%)	25 (83.3%)
Benefit of standardised parent materials^a^	—	173 (77.9%)	24 (80.0%)

*Note:* The longitudinal subgroup (*n* = 30) consists of physicians who participated in both the 2022 baseline survey and the 2025 follow‐up survey. The total number of survey participants in 2025 was 222. Due to occasional missing responses, the number of responses per item may vary.

^a^ Not included in the 2022 survey.

### 3.4 Neurological Assessment and aEEG Interpretation

Regarding the subjective confidence in the interpretation of aEEG findings, 31.8% of the 2022 cohort and 38.3% of the 2025 general cohort reported feeling completely confident. Within the longitudinal subgroup, 60.0% of physicians reported complete subjective confidence in interpreting aEEG findings by 2025. Regarding the neurological assessment of the patient before, during and after TH treatment, 21.2% of the 2022 cohort, 29.3% of the 2025 general cohort and 36.7% of the longitudinal subgroup felt completely confident. Partial confidence regarding neurological assessment was reported by 72.7% of the 2022 cohort, 60.4% of the 2025 general cohort and 63.3% of the longitudinal subgroup (Table 3).

### 3.5 Seizure Management, Communication and Standardised Information

**FIGURE 1:**
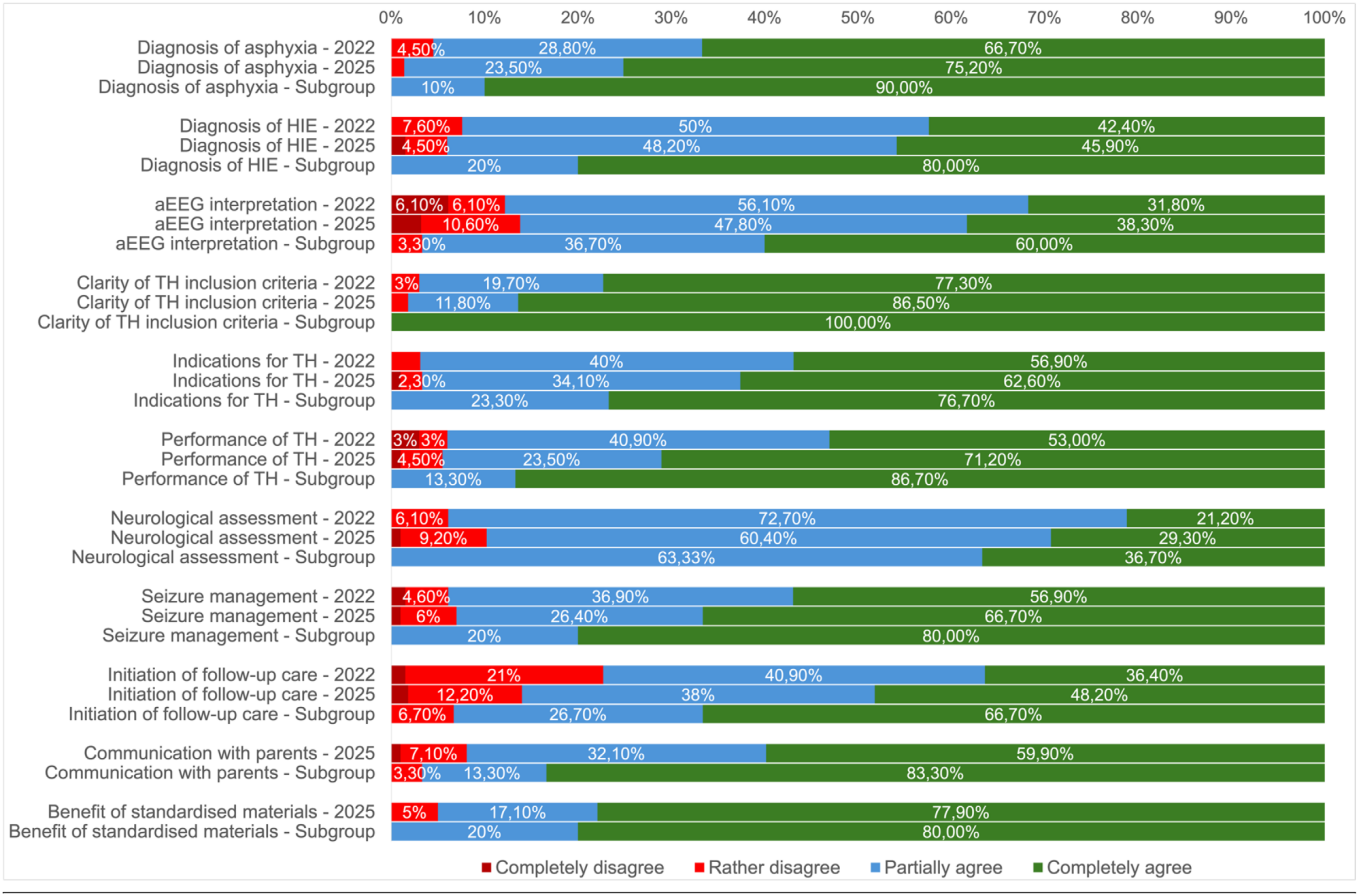
Distribution of confidence levels regarding diagnostic, therapeutic, and communication domains. The longitudinal subgroup (*n* = 30) consists of physicians who participated in both the 2022 baseline survey and the 2025 follow‐up survey.

Subjective confidence in treating seizures during TH treatment subsequently rose, with 56.1% in 2022, 66.7% in the general 2025 cohort and 80.0% in the longitudinal subgroup feeling completely confident. For the initiation of follow‐up care, complete confidence was reported by 36.4% in 2022, 48.2% in 2025 and 66.7% in the longitudinal subgroup. In the 2025 general cohort, 59.9% of physicians felt completely confident in providing parents with clear information regarding treatment, risks and prognosis, while 83.3% of the longitudinal subgroup reported the same. Standardised information materials for parents were requested by 77.9% of the 2025 general cohort and 80.0% of the longitudinal subgroup, who completely agreed that such materials would improve parental education and clinician confidence. Finally, the option of online specialist consultation was supported by 40.9% of the 2022 cohort and 47.3% of the 2025 general cohort. A breakdown of these results is presented in Tables 2 and 3, while Figure 1 illustrates the detailed distribution of confidence levels regarding diagnostic, therapeutic and communication domains.

## 4 Discussion

In this study, we evaluated the professional impact of the German Hypothermia Registry between 2022 and 2025. Our findings demonstrate that sustained Registry participation was associated with an increase in clinical clarity and subjective confidence of physicians across most diagnostic and therapeutic domains, indicating a positive impact of our Registry's efforts. Despite this overall improvement, we identified a persistent bedside confidence gap concerning neurological assessment and aEEG interpretation, even among experienced physicians. Furthermore, while confidence in parental communication was high, an overwhelming demand for standardised educational materials remains.

### 4.1 Professional Maturation and the Registry Effect

The comparative analysis between the baseline cohort in 2022 and the longitudinal subgroup of physicians active through 2025 provides direct insight into the professional impact of the German Hypothermia Registry. The broad expansion of the Registry from 10 initial NICUs to 80 participating NICUs within 3 years reflects a growing national interest and commitment to harmonised care for neonatal HIE. This structural growth was accompanied by a clear professional maturation within the Registry network, evidenced by the predominance of consultants in neonatology and department chiefs.

The longitudinal analysis offers the strongest evidence for a true evaluation of the Registry's impact. Sustained participation in the Registry was associated with a marked increase in clarity and confidence across nearly all diagnostic and therapeutic domains. Most notably, complete clarity regarding inclusion criteria for TH reached 100% among longitudinal participants in 2025, compared to 77.3% in the total cohort at baseline. Similarly, confidence in diagnosing HIE nearly doubled, rising from 42.4% in 2022 to 80.0% in the longitudinal subgroup. These findings suggest that the Registry has successfully implemented national recommendations into routine institutional practice, directly addressing the heterogeneity and uncertainty previously documented in German neonatal HIE care [12].

### 4.2 Persistent Diagnostic Uncertainty and Bedside Confidence Gap

Despite these positive developments, the survey reveals a persistent gap between technical clarity and bedside clinical confidence, particularly in neurological assessment and aEEG interpretation. While confidence in aEEG interpretation nearly doubled from 31.8% in 2022 to 60.0% among longitudinal participants, a substantial proportion of experienced physicians continued to report only partial confidence. This uncertainty was even more pronounced in comprehensive neurological assessment before, during, and after TH, where only 36.7% of longitudinal participants felt completely confident in 2025.

This finding is of particular relevance, as both abnormal aEEG patterns and neurological assessment represent key determinants for initiating TH within the Registry's own admission analysis [9]. The discrepancy between high confidence in protocol‐driven decisions and lower confidence in clinical interpretation is highly relevant. It points out that HIE management cannot be reduced to standardised rules alone, particularly in decentralised care systems with low individual case volumes.

The data therefore suggest that while the Registry has successfully standardised the know‐how of TH, the execution of neurological diagnosis remains the most challenging aspect of HIE care. Even among highly experienced physicians, neurological assessment and aEEG interpretation continue to represent critical areas of uncertainty, defining a central task for future Registry initiatives.

### 4.3 Communication and the Demand for Standardised Information

In addition to diagnostic and therapeutic competencies, the Registry positively influenced clinicians' confidence in managing the broader clinical course of HIE. Confidence in seizure treatment and follow‐up initiation improved substantially over time, with physicians in the longitudinal subgroup reporting high confidence in communicating with parents regarding treatment, risks and prognosis.

Notably, however, high individual confidence did not lessen the perceived need for further standardisation. On the contrary, an overwhelming majority of clinicians strongly supported the development of standardised information materials for parents. These findings reinforce the notion that harmonisation efforts must extend beyond protocols to include educational resources.

### 4.4 Strengths and Limitations

The primary strength of this study lies in its nationwide, longitudinal design, which allows for the direct observation of the Registry's impact over a three‐year period following its implementation. Furthermore, the inclusion of physicians across all professional levels provides a comprehensive overview of the clinical reality in German NICUs and HIE care.

However, several limitations must be considered when interpreting these findings. First, as both the German Hypothermia Registry and the associated surveys rely on voluntary participation, a selection bias cannot be entirely excluded. Second, the interpretation of neurological findings and aEEG remains complex and subject to variability, which further supports the need for ongoing training efforts.

Third, the survey instrument underwent minor modifications between 2022 and 2025 to reflect evolving clinical and educational needs. Certain items, including parental communication and leadership roles such as department chiefs, were only introduced in the 2025 survey. Finally, categorisation of personal case volumes was refined in the second survey, which requires cautious interpretation of these comparative trends.

## 5 Conclusion

This study found that the German Hypothermia Registry contributed substantially to the harmonisation of neonatal HIE care and to increased clinician confidence nationwide. While institutional standardisation and general clinical confidence improved markedly, this study identified a remaining gap in neurological assessment and aEEG interpretation. Bridging this gap will be a key priority for the next phase of the Registry. This requires widening already existing educational formats, such as structured teaching, digital decision‐support tools and supervised training, to translate guideline knowledge into reliable bedside expertise.

## Author Contributions

**Till Dresbach:** conceptualization, investigation, methodology, writing – review and editing, resources, supervision, data curation, project administration, formal analysis, visualization. **Khaled Abdelrahman:** writing – original draft, writing – review and editing, formal analysis, data curation. **Anne Groteklaes:** writing – review and editing. **Hemmen Sabir:** conceptualization, investigation, writing – review and editing, methodology, supervision, resources, data curation, project administration, formal analysis, visualization. **Andreas Mueller:** writing – review and editing. **Sebiha Demir:** writing – review and editing. **Alexandra Boeckenhoff:** writing – review and editing.

## Funding

The authors have nothing to report.

## Conflicts of Interest

The authors declare no conflicts of interest.

## Supporting information


**Appendix S1:** Neonatal hypoxic–ischemic encephalopathy care in Germany: A nationwide two‐phase survey of the German Hypothermia Registry.

**Appendix S2:** Study Group “German Hypothermia Registry”.

## Data Availability

The data that support the findings of this study are available from the corresponding author upon reasonable request.
