# Supplementary material for: German Hypothermia Registry Improves Clinician Confidence and Harmonises Decentralised Neonatal Hypoxic‐Ischaemic Encephalopathy Care in Germany
Source: Acta Paediatr. 2026 May 8;115(9):1910–6. doi: 10.1111/apa.70579 (PMC13460595; doi:10.1111/apa.70579)
Supplement: Supplementary file 1 [file APA-115-1910-s001.docx]

Neonatal hypoxic–ischemic encephalopathy care in Germany: A nationwide two-phase survey of the German Hypothermia Registry (Supporting information Appendices_ Appendix S1)

**German Hypothermia Registry survey of 2022**

1. Hypothermia treatment is carried out at our clinic in accordance with
a) National guidelines of the German association of scientific medical societies
b) Clinic-specific standard operating procedures
c) Individual treatment instruction by senior colleague
d) I don't know

2. I feel confident about the criteria for diagnosing asphyxia.
a) I completely agree
b) I partially agree
c) I rather disagree
d) I completely disagree

3. I feel confident about the criteria for diagnosing encephalopathy.
a) I completely agree
b) I partially agree
c) I rather disagree
d) I completely disagree

4. I feel confident about the findings of the aEEG.
a) I completely agree
b) I partially agree
c) I rather disagree
d) I completely disagree
e) I have not yet performed it myself

5. The inclusion criteria for hypothermia treatment are clear to me.
a) I completely agree
b) I partially agree
c) I rather disagree
d) I completely disagree

6. I feel confident about the indications for performing hypothermia treatment.
a) I completely agree
b) I partially agree
c) I rather disagree
d) I completely disagree

7. I feel confident about performing hypothermia treatment.
a) I completely agree
b) I partially agree
c) I rather disagree
d) I completely disagree

8. I feel confident in my neurological assessment of the patient before, during, and after hypothermia treatment.
a) I completely agree
b) I partially agree
c) I rather disagree
d) I completely disagree

9. I feel confident in treating possible seizures that may occur during hypothermia treatment.
a) I completely agree
b) I partially agree
c) I rather disagree
d) I completely disagree

10. I feel confident in initiating follow-up care for patients after hypothermia treatment.
a) I completely agree
b) I partially agree
c) I rather disagree
d) I completely disagree

11. I would like to see a national, uniform protocol with standardised treatment recommendations.
a) I completely agree
b) I partially agree
c) I rather disagree
d) I completely disagree

12. I would like to have the option of consulting with specialists online.
a) I completely agree
b) I partially agree
c) I rather disagree
d) I completely disagree

13. How many patients receiving hypothermia treatment have you personally supervised, accompanied, or treated in the last 12 months?

a) 0
b) 1-5
c) >5

14. What is your current position at the clinic?
a) Paediatric resident
b) Fellow in neonatology
c) Consultant in neonatology

15. Work experience within neonatology
a) 0-1 year
b) 2-4 years
c) 5 years or more

16. Volume of employment
a) Full-time
b) Part-time

17. Gender
a) Female
b) Male
c) Diverse
d) Not specified

18. How old are you?
a) 25–29 years
b) 30–39 years
c) 40–49 years
d) 50–59 years
e) 60 years or older
f) No answer

**German Hypothermia Registry survey of 2025**

1. Hypothermia treatment is carried out at our clinic in accordance with
a) National guidelines of the German association of scientific medical societies
b) Clinic-specific standard operating procedures
c) Individual treatment instruction by senior colleague
d) I don't know

2. I feel confident about the criteria for diagnosing asphyxia.
a) I completely agree
b) I partially agree
c) I rather disagree
d) I completely disagree

3. I feel confident about the criteria for diagnosing encephalopathy.
a) I completely agree
b) I partially agree
c) I rather disagree
d) I completely disagree

4. I feel confident about the findings of the aEEG.
a) I completely agree
b) I partially agree
c) I rather disagree
d) I completely disagree
e) I have not yet performed it myself

5. The inclusion criteria for hypothermia treatment are clear to me.
a) I completely agree
b) I partially agree
c) I rather disagree
d) I completely disagree

6. I feel confident about the indications for performing hypothermia treatment.
a) I completely agree
b) I partially agree
c) I rather disagree
d) I completely disagree

7. I feel confident about performing hypothermia treatment.
a) I completely agree
b) I partially agree
c) I rather disagree
d) I completely disagree

8. I feel confident in my neurological assessment of the patient before, during, and after hypothermia treatment.
a) I completely agree
b) I partially agree
c) I rather disagree
d) I completely disagree

9. I feel confident in treating possible seizures that may occur during hypothermia treatment.
a) I completely agree
b) I partially agree
c) I rather disagree
d) I completely disagree

10. I feel confident in initiating follow-up care for patients after hypothermia treatment.
a) I completely agree
b) I partially agree
c) I rather disagree
d) I completely disagree

11. I would like to see a national, uniform protocol with standardised treatment recommendations.
a) I completely agree
b) I partially agree
c) I rather disagree
d) I completely disagree

12. I would like to have the option of consulting with specialists online.
a) I completely agree
b) I partially agree
c) I rather disagree
d) I completely disagree

13. Standardised treatment recommendations would make my daily work easier and give me more confidence when performing hypothermia treatment.
a) I completely agree
b) I partially agree
c) I tend to disagree
d) I completely disagree

14. I feel confident in providing parents with clear information about hypothermia treatment, the procedure involved, and the possible risks and prognosis.
a) I completely agree
b) I partially agree
c) I tend to disagree
d) I completely disagree

15. Standardised information materials for parents would improve education and give me more confidence in communication.

a) I completely agree
b) I partially agree
c) I tend to disagree
d) I completely disagree

16. How many patients receiving hypothermia treatment have you personally supervised, accompanied, or treated in the last 12 months?

a) 1
b) 2
c) 3
d) 4
e) 5
f) 6
g) 7 or more

17. What is your current position at the clinic?
a) Paediatric resident
b) Fellow in neonatology
c) Consultant in neonatology
d) Department chief
e) Other (please specify)

18. Work experience within neonatology
a) 0-1 year
b) 2-4 years
c) 5 years or more

19. Volume of employment
a) Full-time
b) Part-time

20. Gender
a) Female
b) Male
c) Diverse
d) Not specified

21. How old are you?
a) 25–29 years
b) 30–39 years
c) 40–49 years
d) 50–59 years
e) 60 years or older
f) No answer

22. Have you participated in the previous survey of the Hypothermia Registry?
a) Yes
b) No
