# Supplementary material for: German Hypothermia Registry Improves Clinician Confidence and Harmonises Decentralised Neonatal Hypoxic‐Ischaemic Encephalopathy Care in Germany
Source: Acta Paediatr. 2026 May 8;115(9):1910–6. doi: 10.1111/apa.70579 (PMC13460595; doi:10.1111/apa.70579)
Supplement: Supplementary file 2 [file APA-115-1910-s002.docx]

Study Group “German Hypothermia Registry”

(Supporting information Appendices_ Appendix S2)

**Appendix S2: Members of the Hypothermia Registry Group
Description:** This table lists the contributing study group members and their respective hospitals who participate in the German Hypothermia Registry.

| **Hospital Names** | **Study Group Members** |
| --- | --- |
| University Hospital **Aachen** | Dr. med. Milian Brasche |
| University Hospital **Augsburg** | Dr. med. Nicolas Allgaier |
| University Hospital OWL **Bielefeld** | Prof. Dr. med. Daniel Klotz |
| **Böblingen** Hospital | Dr. med. Feldhahn  Dr. med. Nachtrodt |
| University Hospital Bochum | Dr. med. Norbert Teig |
| Marien Hospital **Bonn** | PD Dr. med. Katja Schneider |
| University Hospital **Bonn** | Prof. Dr. med. Hemmen Sabir |
| University Hospital **Brandenburg an der Havel** | Sandra Holz |
| Children's Hospital **Bremen** | Dr. med. Birte Tröger  Dr. med. Thorsten Körner (zuletzt!) |
| **Chemnitz** Hospital | Uwe Höpner |
| St. Vinzenz Hospital **Dinslaken** | Marie Schmenk |
| **Dortmund** Hospital | Dr. med. Michael Hofmann |
| University Hospital **Dresden** | Prof. Dr. med. Mario Rüdiger |
| Sana Hospital **Duisburg** | Dr. med. Julia Osterholt |
| Florence Nightingale Hospital, Department of Paediatrics **Düsseldorf** (Kaiserswerth) | Dr. med. Martin Berghäuser |
| University Hospital **Düsseldorf** | Dr. med. Christopher Willerscheidt |
| Evangelical Hospital **Düsseldorf** | Farina Morgenroth |
| Helios Hospital **Erfurt** | Dr. med. Hans Jörg Bittrich |
| University Hospital **Erlangen** | Dr. Steven Hébert |
| Elisabeth Hospital **Essen** | Arber Arapi |
| University Hospital **Essen** | Anja Stein |
| **Esslingen** Hospital | Dr. med. Veit Lange |
| DIAKO Hospital **Flensburg** | Dr. med. Gunnar Rau |
| Bürgerhospital **Frankfurt** | Prof. Dr. med. Kunzmann |
| University Hospital **Frankfurt** | Dr. med. Nikolas Hillenbrandt |
| **Frankfurt** Höchst Hospital | Dr. med. Matthias Reger |
| St. Clemens Hospital **Geldern** | Susanne Schade |
| University Hospital **Göttingen** | PD Dr. Jana Dieks |
| University Hospital **Greifswald** | Dr. med. Heimke von Osten |
| AGAPLESION Hospital **Hagen** | Dr. med. Marita Horstkemper |
| AKK Children's Hospital  (**Hamburg Altona**) | PD Dr. med. Martin Blohm |
| University Hospital **Hamburg-Eppendorf** | Dr. med. Mario Lange |
| **Hannover** Medical School | Prof. Dr. med Bettina Bohnhorst |
| University Hospital **Heidelberg** | Dr. med. Simon Kranig |
| SLK-Hospitals **Heilbronn** | Dr. med. Maurice Petrasch |
| University Hospital of Saarland  (**Homburg**) | PD Dr. med. Nasenien Nourkami-Tutdibi |
| University Hospital **Jena** | Dr. med. Christiane Pinkwart |
| Westpfalz Hospital **Kaiserslautern** | PD Dr. med. Thomas Kriebel |
| Municipal Hospital **Karlsruhe** | Dr. med. Julia Jerabek |
| Children's Hospital   Amsterdamer Str. **Köln** | Dr. med. Nora Bonney |
| University Hospital **Köln** | Prof. Dr. med. Angela Kribs |
| Helios Hospital **Krefeld** | Clemens Andrée |
| Children's Hospital  St. Marien gGmbH **Landshut** | Sophia Stadermann |
| University Hospital **Leipzig** | PD Dr. med. Kirsten Glaser |
| **Leverkusen** Hospital | Dr. med. Peter Jahn |
| RKH Hospital **Ludwigsburg** | Dr. med. Katrin Braun  Dr. med. Anna Eisenhardt |
| University Hospital **Mannheim** | Dr. med. Alba Perez-Ortiz |
| University Hospital **Marburg** | PD Dr. med. Nadine Mand |
| Johannes Wesling Hospital **Minden** | Dr. med. Timea Takacs |
| **Mönchengladbach** Hospital | Dr. med. Anja Jäger (Oberärztin)  Dr. med. Oliver Schroers (Oberarzt) |
| LMU University Hospital **Munich** | Dr. med. Esther Schouten (Großhadern) Prof. Dr. med. Claudia Nussbaum, (Innenstadt) |
| St. Franziskus Hospital **Münster** | Dr. med. Kai Böckenholt |
| Rheinland Hospital  Lukaskrankenhaus **Neuss** | Rüdiger Wentzell  Prof. Dr. med. Guido Engelmann |
| Marienhaus Hospital St. Elisabeth **Neuwied** | Dr. med. Katherine Louise Kastor |
| Hallerwiese-Cnopfsche  Children's Hospital **Nürnberg** | Prof. Dr. med. Michael Schroth |
| Sana Hospital **Offenbach** | Dr. med. Matthias Engler |
| University Hospital **Oldenburg** | Dr. med. Friederike Eichhorn |
| Children's Hospital **Ortenau** | Dr. med. Moritz Rohrbach |
| Christian Children's Hospital **Osnabrück** | PD Dr. med. Fabian Bärtling |
| St. Vincenz Hospital **Paderborn** | Dr. med. Frank Dohle |
| Children's Hospital Dritter Orden **Passau** | Prof. Dr. med. Matthias Keller |
| Helios Hospital **Pforzheim** | Marion Weber |
| Hospital Barmherzige Brüder  **Regensburg** | Dr. med Verena Lehnerer |
| District Hospital **Reutlingen** | Dr. med Anja Mayer |
| **Saarbrücken** Hospital | Dr. med. Marie-Claire Detemple |
| Asklepios Children's Hospital **Sankt Augustin** | Dr. med. Valentin Oellers |
| DRK Children's Hospital **Siegen** | Markus Pingel |
| Hegau-Bodensee Hospital **Singen** | Dr. med. Friederike Eilbracht |
| Municipal Hospital **Solingen** | Dr. med. Jutta Adler |
| Diakonissen Foundation Hospital **Speyer** | Elena Eberle |
| SRH Central Hospital **Suhl** | Karoline Goj |
| **Traunstein** Hospital | Dr. med. Virginia Toth |
| Hospital Mutterhaus der Borromäerinnen **Trier** | Andrea Czoske |
| University Hospital **Tübingen** | Dr. med. Cornelia Wiechers |
| St. Marien Hospital **Vechta** | Christiane Mayer |
| Schwarzwald-Baar Hospital **Villingen-Schwenningen** | Dr. med. Anja Schumann |
| Marien Hospital **Wesel** | Patrick Salaschek |
| Rems-Murr Hospital **Winnenden** | Dr. med. Janaina Rauch |
| Helios University Hospital **Wuppertal** | Prof. Dr. med. Kai Hensel |
| University Hospital **Würzburg** | Dr. med. Pia Paul |
